# Metabolic dysfunction-associated fatty liver disease in chronic hepatitis B: dual effects on hepatocarcinogenesis and evolving strategies for risk prediction

**DOI:** 10.3389/fimmu.2026.1817642

**Published:** 2026-06-12

**Authors:** Huihui Tao, Yuxin Duan, Guoyang Yu, Wei Yan, Jianhua Yin

**Affiliations:** 1 Department of Laboratory Medicine, Naval Medical Centre, Naval Medical University, Shanghai, China; 2 Department of Epidemiology, Naval Medical University, Shanghai, China; 3 Key Laboratory of Biological Defense, Ministry of Education, Naval Medical University, Shanghai, China; 4 Shanghai Key Laboratory of Medical Bioprotection, Naval Medical University, Shanghai, China

**Keywords:** chronic hepatitis B, comorbidity management, hepatocellular carcinoma, metabolic dysfunction-associated fatty liver disease, molecular mechanisms, risk prediction models

## Abstract

**Aim:**

Chronic hepatitis B (CHB) remains a major global cause of hepatocellular carcinoma (HCC), particularly in regions with high hepatitis B virus endemicity. Meanwhile, metabolic dysfunction-associated fatty liver disease (MAFLD) has emerged as a rapidly growing metabolic contributor to hepatocarcinogenesis. Their co-occurrence influences liver cancer development through complex biological interactions. This review aims to summarize recent advances regarding how CHB–MAFLD comorbidity affects HCC risk and the development of related predictive models.

**Methods:**

This review synthesizes evidence from recent clinical and basic research exploring the relationship between CHB–MAFLD comorbidity and cancer risk. It evaluates the performance of traditional and next-generation risk prediction models and examines the potential of artificial intelligence in integrating multimodal data to improve accuracy.

**Results:**

The underlying pathophysiology exhibits a dual nature. While metabolic disturbances can suppress hepatitis B virus replication and facilitate surface antigen clearance, metabolic dysfunctions such as obesity and diabetes independently accelerate fibrosis and increase cancer risk. This paradox originates from heterogeneous effects: simple steatosis may reduce risk, whereas cumulative metabolic abnormalities increase risk in a dose-dependent manner. Although traditional models retain prognostic value, their sensitivity is suboptimal. Next-generation models incorporating metabolic parameters have demonstrated improved accuracy. Furthermore, machine learning shows considerable potential for modeling complex non-linear interactions and enabling individualized risk prediction.

**Conclusion:**

This review summarizes how metabolic dysfunction-associated fatty liver disease influences cancer risk in patients with chronic hepatitis B. It provides a framework for identifying high-risk individuals to guide stratified, precision management strategies.

## 1 Introduction

Hepatocellular carcinoma (HCC) ranks as the third leading cause of cancer-related mortality worldwide. It is frequently diagnosed at an advanced stage, when curative treatments are limited. This late detection contributes to a dismal five-year relative survival rate of below 20%, a median survival of under one year, and a significantly impaired quality of life (1, 2). This clinical reality not only imposes a substantial individual disease burden but also underscores the limitations of current diagnostic and therapeutic strategies, positioning HCC as a critical public health issue requiring urgent attention.

Chronic hepatitis B virus (HBV) infection is a primary global etiology of HCC, particularly in the Asia-Pacific region (3). An estimated 296 million individuals worldwide live with chronic HBV infection, which leads to approximately 820,000 deaths annually, primarily from cirrhosis and HCC (4, 5). Notably, China bears a disproportionately high burden of HBV infection and HBV-related HCC because of both the historically high prevalence of chronic HBV infection and its large population base. Although universal HBV vaccination and antiviral therapy have substantially reduced HBV-related morbidity and mortality, HBV infection remains a dominant contributor to HCC incidence in China (6).

However, the etiological landscape of HCC is undergoing a profound shift. In the classic paradigm of HBV-related hepatocarcinogenesis, metabolic cofactors have long been under-recognized. Driven by the obesity epidemic, metabolic dysfunction-associated fatty liver disease (MAFLD) has emerged as the fastest growing cause of HCC in Western populations, conferring a significantly elevated oncogenic risk even in non-cirrhotic individuals (7, 8). Emerging evidence now demonstrates that MAFLD-derived metabolic stress, such as oxidative injury and mitochondrial dysfunction, intertwines with chronic HBV-induced damage. Mechanisms like HBx protein-driven oncogenic signaling may act in concert to promote the malignant transformation of hepatocytes (7, 8).

MAFLD was defined in 2020 as an extension of non-alcoholic fatty liver disease (NAFLD), aiming to more explicitly acknowledge the central role of metabolic dysfunction (9, 10). In contrast to NAFLD, MAFLD is diagnosed based on the presence of hepatic steatosis concurrent with overweight or obesity, type 2 diabetes, or at least two metabolic risk abnormalities, and other liver diseases need not be excluded (9, 11, 12). This inclusive diagnostic framework aligns more closely with clinical practice and is particularly valuable for assessing patients with comorbid conditions.

The co-occurrence of CHB and MAFLD has become increasingly common, representing a defining feature of contemporary HCC epidemiology. A meta-analysis of 48,472 patients reported a global CHB–MAFLD co-prevalence of 34.9%, revealing a far greater metabolic burden in viral hepatitis patients than previously recognized (13). Model-based projections forecast a sharp rise in MAFLD-related HCC incidence from 2016 to 2030 in several countries, with China projected to experience the most dramatic increase from 14,090 to 26,240 cases (6, 14). This evolving epidemiological landscape is particularly relevant in China, where the persistent burden of HBV-related liver disease increasingly overlaps with the rapidly rising prevalence of metabolic dysfunction. Emerging evidence indicates that CHB–MAFLD comorbidity not only accelerates liver disease progression but also leads to later-stage HCC diagnosis, poorer prognosis, and more limited therapeutic options, thereby exacerbating the overall disease burden (15, 16).

Nevertheless, the impact of MAFLD on HCC risk in CHB patients remains controversial. Some cohort studies indicate that MAFLD independently elevates HCC risk, particularly in non-cirrhotic populations (8, 17, 18). Conversely, other investigations suggest that simple hepatic steatosis may reduce HCC risk by suppressing HBV replication, with this protective effect reversing in the presence of metabolic abnormalities like diabetes (16, 19). Recent severity-stratified analyses further suggest that the biological and clinical impact of MAFLD in CHB patients may depend on the degree of hepatic steatosis and associated metabolic dysfunction, with moderate-to-severe MAFLD exerting more pronounced adverse effects on antiviral treatment outcomes and clinical prognosis than mild steatosis (20). These discrepancies may stem from the evolution of diagnostic criteria (from exclusive NAFLD to inclusive MAFLD), heterogeneity among study populations, and the differential effects of specific metabolic components (9, 21). Furthermore, established HCC risk prediction models, such as REACH-B and PAGE-B, are primarily based on virological or clinical parameters and largely fail to incorporate metabolic factors, limiting their predictive performance in comorbid patients (22).

This review synthesizes current evidence on the molecular mechanisms, risk prediction models, and clinical management of CHB–MAFLD comorbidity. It aims to provide a theoretical framework for refining HCC risk stratification and developing individualized prevention strategies for this high-risk population, thereby advancing precision medicine for patients with this comorbidity.

## 2 Etiology and synergistic pathogenic mechanisms

Investigating the mechanisms underlying CHB–MAFLD comorbidity reveals a complex interplay. This complexity manifests as discrepancies between clinical and basic research findings, short- and long-term outcomes, and virological versus histological parameters. It reflects the liver’s unique dual role in metabolism and immunity, suggesting that understanding their interactions requires moving beyond a simplistic “promotion versus inhibition” model toward a dynamic, phase-dependent integrative framework.

### 2.1 The contradictory nature of CHB–MAFLD interaction mechanisms

Early observational studies suggested a potentially reciprocal inhibitory relationship between MAFLD and CHB during disease progression (23). Epidemiological data indicate that MAFLD prevalence among CHB patients is approximately 30%, lower than in the general population, initially supporting a hypothesis that HBV infection might exert a “protective effect” against fatty liver disease (11, 24). The Taiwanese REVEAL-HBV cohort study reported that obese CHB patients (BMI ≥ 30 kg/m²) had a significantly higher HBsAg seroclearance rate than non-obese patients [risk ratio (RR) = 1.51], with serum HBV DNA levels inversely correlated with waist circumference and triglyceride levels (25). Similarly, a study involving 3,212 untreated CHB patients showed that hepatic steatosis—a core pathological feature of MAFLD—was associated with reduced intrahepatic hepatitis B core antigen (HBcAg) expression (26). A meta-analysis further confirmed an inverse association between hepatic steatosis and HBV viral activity, suggesting that metabolic dysregulation may partially suppress HBV replication (27).

However, subsequent in-depth studies have elucidated a more intricate nature of their interaction. Several prospective cohorts have demonstrated that, although MAFLD may transiently suppress HBV replication, its coexistence with CHB is an independent risk factor for HCC, leading to a markedly higher HCC incidence compared to CHB alone (11, 28). The fundamental mechanisms behind this paradox may involve two principal aspects. Firstly, synergistic genomic damage induced by viral and metabolic factors—HBV DNA integration exhibits site-specific genomic preferences, while MAFLD-associated oxidative stress exacerbates genomic instability, collectively promoting hepatocyte malignant transformation (29, 30). Secondly, microenvironmental dysfunction from immune–metabolic reprogramming—under chronic viral infection and in tumor conditions, CD8^+^ T cells typically exhibit an exhausted phenotype with high PD-1 expression. Their impaired mitochondrial function and reduced oxidative phosphorylation capacity are key mechanisms underlying defective antitumor immunity (31). In the MAFLD–CHB comorbid state, increased genomic instability and impaired immune surveillance are expected to exert additive effects, jointly facilitating tumor immune escape. In summary, intrinsic genetic injury in hepatocytes coupled with dysregulation of the tumor immune microenvironment forms the pathological basis for the interaction between metabolic factors and HBV infection, synergistically amplifying HCC risk.

### 2.2 Toward a unified understanding of CHB–MAFLD interaction mechanisms

Recent evidence suggests that the interaction between HBV and MAFLD/NAFLD represents a highly dynamic and complex process. Its ultimate clinical and biological manifestations depend on two key factors. The first is the severity of metabolic abnormalities, such as the degree of steatosis and the number of metabolic syndrome components. The second determinant is the evolving disease phase (e.g., immune-tolerant *vs.* immune-active stages) and the surrounding pathological microenvironment (4, 19).

It is important to distinguish simple hepatic steatosis from metabolically active steatohepatitis/NASH. Simple steatosis mainly reflects lipid accumulation and may be associated with reduced HBV replication in some studies, whereas steatohepatitis/NASH represents a more advanced inflammatory and lipotoxic state characterized by hepatocellular injury, oxidative stress, mitochondrial dysfunction, and fibrogenic activation. At the molecular level, NASH is associated with stronger activation of NF-κB, JNK, and inflammasome signaling, together with more pronounced impairment of autophagy and mitochondrial quality control than simple steatosis. Therefore, these two conditions may have distinct biological and prognostic implications in CHB–MAFLD comorbidity (16, 19, 20).

During early disease stages or with mild hepatic steatosis, the metabolic milieu may exert transient antiviral effects via innate immunity activation. Studies show that lipid metabolites like free fatty acids (FFAs) act as endogenous ligands, activating Toll-like receptor 4 (TLR4) on hepatocytes and immune cells. This triggers the myeloid differentiation primary response 88 (MyD88)-dependent signaling pathway, leading to the production of proinflammatory cytokines such as type I interferons (e.g., IFN-β) and interleukin-6 (IL-6) (32). These cytokines have been shown to suppress HBV replication, potentially explaining the lower viral loads observed in some comorbid patients. In advanced steatohepatitis/NASH, persistent metabolic dysregulation promotes excessive accumulation of lipotoxic species (33). These lipotoxic mediators, including free cholesterol and ceramides, induce pronounced endoplasmic reticulum stress and mitochondrial dysfunction, culminating in hepatocellular injury and death (34). At this stage, a synergistic interaction emerges between metabolic stress and the pathogenic activities of HBV viral proteins, particularly HBx. The HBx protein disrupts mitochondrial function and promotes oxidative stress, thereby potentiating NASH-associated lipotoxicity. Together, these processes exacerbate metabolic dysfunction, inflammation, and hepatic stellate cell activation, accelerating liver fibrosis and hepatocarcinogenesis (4, 35).

**Figure 1:**
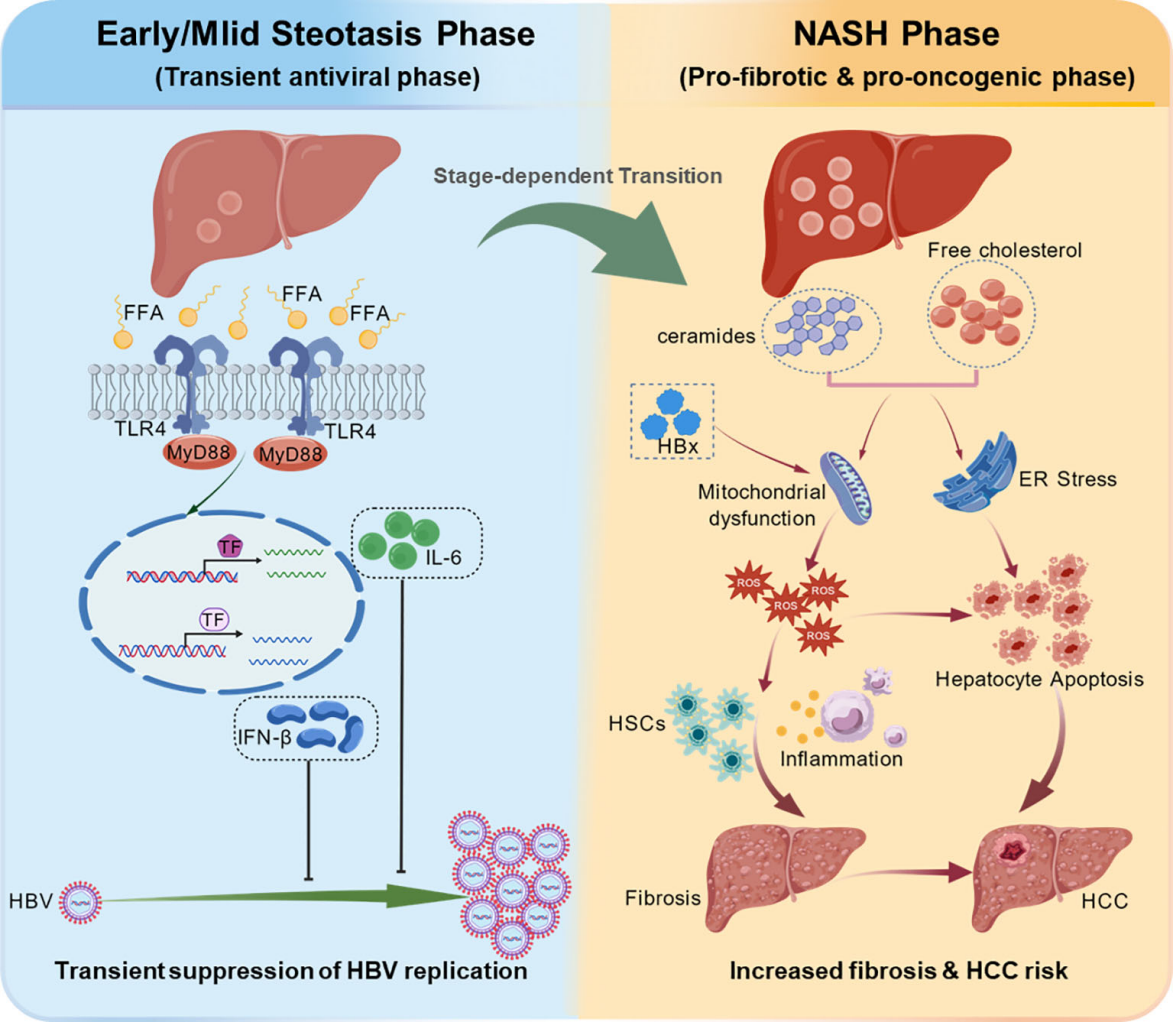
Stage-dependent bidirectional crosstalk between HBV and MAFLD in liver disease progression. In early stages, metabolic activation and innate immune signaling may transiently suppress HBV replication. In advanced stages, lipotoxicity, endoplasmic reticulum stress, and HBx-mediated oxidative injury synergistically promote hepatic inflammation, fibrosis, and malignant transformation. FFA, fatty acids; TLR4, toll-like receptor 4; MyD88, myeloid differentiation primary response 88; TF, Transcription Factor; IL6, interleukin-6; IFN-β, Type I interferon; HBV, hepatitis B virus; NASH, nonalcoholic steatohepatitis; ER, endoplasmic reticulum; HBx, hepatitis B virus X protein; HSC, Hepatic Stellate Cell; ROS, reactive oxygen species; HCC, hepatocellular carcinoma.

Thus, the impact of MAFLD/NAFLD on CHB appears to evolve dynamically during disease progression in a bidirectional and stage-dependent manner. In the early phase, mild steatosis and metabolic activation may transiently suppress HBV replication through innate immune activation and inflammatory cytokine signaling. However, as metabolic dysfunction progresses toward steatohepatitis and systemic metabolic derangement, the dominant biological effects gradually shift. Persistent lipotoxicity, oxidative stress, mitochondrial dysfunction, and chronic inflammation progressively outweigh the transient antiviral effects and establish a pro-fibrotic, pro-inflammatory, and immunosuppressive microenvironment. In this setting, HBV-related oncogenic mechanisms, particularly HBx-mediated oxidative injury and immune dysregulation, may synergize with metabolic stress to accelerate hepatocarcinogenesis. This stage-dependent framework provides a mechanistic explanation for the seemingly paradoxical finding that hepatic steatosis may transiently suppress HBV replication, whereas CHB–MAFLD comorbidity remains linked to increased long-term risks of fibrosis and HCC, as illustrated in **Figure 1**.

## 3 Hepatocellular carcinoma risk prediction models: from traditional scoring to machine learning

Accurate HCC risk prediction is fundamental for the personalized management of chronic liver disease. Robust risk assessment and stratification of high-risk populations are pivotal for enabling early detection, optimizing surveillance, guiding individualized interventions, and ultimately improving outcomes. In recent years, HCC risk prediction models have undergone a notable paradigm shift—from traditional clinical parameter-based scoring systems to precision models powered by artificial intelligence (AI) and machine learning (ML) that integrate multimodal data. This evolution has contributed to improved predictive performance and translational potential (22, 36, 37). **Table 1** provides a comparative summary of traditional and ML–driven HCC risk prediction models, highlighting their target populations, key variables, and performance.

**Table 1:** Comparative summary of traditional and machine learning–based models for HCC risk prediction.

Model	Year	Country/area	Age	Sex	Ther-apy	Cirrhos-is	Laboratory variables						Metabolic factors				Others	AUROC	Validation
							HBeAg	HBV	ALT	PLT	ALB	AFP	DM	BMI	HT	CHOL			
REACH-B	2011	Taiwan	**√**	**√**			**√**	**√**	**√**									0.796	✓
CU-HCC	2010	HongKong	**√**			**√**		**√**			**√**						BIL	0.76	✓
GAG-HCC	2009	HongKong	**√**	**√**		**√**		**√**									CPM	0.88	
PAGE-B	2016	Greece	**√**	**√**	**√**					**√**								0.82	✓
mPAGE-B	2018	Korea	**√**	**√**	**√**					**√**	**√**							0.82	✓
aMAP	2020	China	**√**	**√**	**√**					**√**	**√**							0.85	✓
CAMD	2018	Taiwan	**√**	**√**	**√**	**√**							**√**					0.82	✓
REAL-B	2020	Asia-Pacific	**√**	**√**	**√**	**√**				**√**		**√**	**√**				DH	0.80	✓
HCC-RIFLE	2024	Korea	**√**	**√**					**√**				**√**	**√**			GGT	0.72	✓
PPDHG	2023	China					**√**			**√**			**√**				PT INR, GLB	0.82	
Lee-ML	2023	Korea	**√**	**√**	**√**	**√**	**√**	**√**	**√**	**√**	**√**	**√**	**√**		**√**		AST, PT INR, Cr	0.87	✓
PLAN-B-DF	2025	Korea	**√**		**√**		**√**	**√**	**√**	**√**			**√**		**√**		six CT biomarkers	0.91	✓
Sarkar-ML	2024	America	**√**	**√**		**√**					**√**		**√**	**√**	**√**	**√**	ALP, Cr, BUN, Na+, K+, PT INR, Cl-, BIL	0.97	✓

A check mark (✓) indicates that the corresponding variable is included in the model. HBeAg, hepatitis Be antigen; HBV DNA, Hepatitis B virus DNA level; ALT, serum alanine aminotransferase concentration; PLT, platelet; ALB, albumin; AFP, alpha-fetoprotein level; DM, diabetes mellitus; BIL, bilirubin; CPM, core promoter mutation; DH, drinking history; BMI, body mass index; GGT, gamma-glutamyl transferase; PT INR, prothrombin time/international normalized ratio; GLB, globulin; HT, hypertension; AST, aspartate aminotransferase; Cr, serum creatinine level; ALP, alkaline phosphatase; BUN, blood urea nitrogen; Na+, sodium; K+, potassium; Cl^-^, Chloride; CHOL, cholesterol.

For CHB–MAFLD comorbidity, major cohort studies underscore the difficulty of risk prediction because hepatic steatosis and metabolic dysfunction may exert divergent prognostic effects. While simple steatosis may be associated with lower HBV activity and, in some settings, reduced HCC risk (16, 19), metabolic dysfunction, diabetes, obesity, and cumulative metabolic abnormalities are more consistently linked to fibrosis progression and increased HCC risk (7, 17, 18, 21). These findings suggest that simple steatosis and metabolically active dysfunction should not be treated as equivalent risk modifiers, highlighting the need for future HCC prediction models to incorporate refined metabolic phenotyping and dynamic metabolic risk assessment.

### 3.1 Traditional HCC risk scoring systems

Traditional HCC risk scoring systems are primarily developed using classical statistical methods such as Cox proportional hazards regression and logistic regression, typically from retrospective cohort studies. These methods identify independent risk factors and assign linear or non-linear weights to construct simple, interpretable scoring models (38). Due to their convenience and strong clinical applicability, they remain the primary tools recommended by clinical practice guidelines (22).

The earliest models were developed specifically for CHB patients, incorporating demographics, laboratory markers, and clinical history to estimate risk and guide follow-up (3). Models like REACH-B score were simple but often underestimated risk in the highest-risk cohorts by omitting cirrhosis (39). This limitation prompted the development of the CU-HCC score, which incorporated cirrhosis to enhance risk stratification for patients with impaired liver function (40). A subsequent advance was the GAG-HCC score, which introduced a molecular marker (HBV core promoter mutation), marking an initial shift toward molecular risk prediction, though its broader application was limited by non-standardized testing (41).

A major limitation of early scores was their inability to account for dynamic risk changes following antiviral therapy. While the nucleotide analogues (NAs) substantially reduced HCC risk, they did not eliminate it, prompting the development of new models for treated patients. The PAGE-B score, incorporating age, sex, and platelet count became a key recommendation in the European Association for the Study of the Liver (EASL) guidelines for on-therapy risk assessment (42). It was later refined to mPAGE-B score with the addition of albumin to better assess hepatic functional reserve (43). However, the persistent residual risk in cirrhotic patients, even with low scores, underscored the need for vigilant monitoring.

The latest evolution in this framework has been the integration of metabolic comorbidities. The CAMD score pioneered this by incorporating diabetes, highlighting the independent contribution of metabolic syndrome and providing a valuable reference for managing complex patients (44). Building upon this, the REAL-B score integrated cirrhosis, diabetes, and demographic variables into a more comprehensive framework, demonstrating excellent predictive performance despite slightly greater complexity (45). The evolution—from static to dynamic assessment and from single-etiology (HBV) to a multifactorial integration—has ushered CHB management into a new era where precise prediction and individualized monitoring are equally important priorities.

The dramatic global increase in MAFLD prevalence has established it as a major driver of HCC, creating an urgent need for dedicated prediction tools. Compared to virus-driven HCC, MAFLD-related HCC is closely associated with specific metabolic abnormalities and liver fibrosis progression, necessitating distinct variables. Commonly used liver fibrosis assessment tools such as FIB-4 and NFS are applied for initial risk stratification, as fibrosis is a key pathway to HCC (46, 47). However, models specifically for MAFLD-related HCC prediction remain in the early stages of development. The HCC-RIFLE score represents a major advance as the first HCC prediction tool tailored for non-cirrhotic NAFLD patients, incorporating metabolism-related indicators (age, sex, diabetes, obesity, ALT, and GGT) and demonstrating robust predictive performance (10-year AUROC of 0.75), addressing a critical gap (38).

The increasing prevalence of CHB–MAFLD comorbidity is particularly concerning because it accelerates liver disease progression and substantially elevates HCC risk. Despite this heightened threat, there remains a critical lack of HCC-specific risk prediction tools explicitly designed for this dual-etiology population. Although the recently proposed PPDHG model (Platelets, Prothrombin Time, Diabetes, HBeAg status, Globulin) effectively assesses advanced fibrosis risk, its focus is not direct HCC prediction (48). Moreover, existing cross-etiology models like the aMAP score—while generally applicable—may not capture the unique pathophysiological interactions of CHB–MAFLD comorbidity, fundamentally limiting their precision for this population (49). Therefore, developing specialized HCC risk prediction tools for patients with coexisting CHB and MAFLD remains a critical and urgent challenge in hepatology.

### 3.2 Emerging machine learning and artificial intelligence models

The introduction of ML and AI represents a significant advancement in HCC prediction models. These technologies overcome the linear assumptions of traditional statistics, enabling automated identification of critical features from complex, high-dimensional, non-linear datasets, consistently showing enhanced predictive performance in retrospective studies. For example, an ensemble ML model developed by Lee et al., combining Random Forest, XGBoost, and logistic regression, achieved an area under curve (AUC) of 0.872 in a validation cohort, markedly outperforming traditional models such as CAMD (0.788) and REAL-B (0.801) (50). This model effectively stratified risk by integrating over a dozen clinical indicators and used SMOTE technology to address data imbalance. Furthermore, the PLAN-B model series, particularly PLAN-B-DF, maintained stable performance (C-index 0.89) across ethnic validations by incorporating advanced computed tomography (CT) radiomic features such as liver volume and visceral fat ratio (51, 52). Similarly, in MAFLD, an ML model by Sarkar et al. based on routine metrics like FIB-4 achieved 92.06% accuracy (AUROC 0.97) in external validation, showing excellent discriminatory power, especially for non-cirrhotic patients (36).

Despite their promising predictive performance, ML- and AI-based HCC prediction models still face important barriers to clinical translation. Many existing models are developed from retrospective and highly selected cohorts, which increases the risk of overfitting and limits generalizability across heterogeneous real-world populations (53, 54). In addition, differences in ethnicity, disease etiology, antiviral treatment status, imaging protocols, and data completeness may further reduce external applicability (53). Prospective validation remains insufficient, and the long-term stability, clinical utility, and implementation feasibility of many models have not been fully established (48, 53). Moreover, some models rely on specialized radiomic or genomic inputs that are not routinely available, while “black box” decision-making limits interpretability and clinician trust (53, 55). Future studies should therefore prioritize multicenter prospective validation, standardized reporting, improved interpretability, and the use of clinically accessible variables to support routine implementation.

Despite these limitations, ML–driven predictive models offer a transformative perspective for liver cancer prevention. They reflect a paradigm shift in hepatology toward data-driven, intelligent management, facilitating the transition from basic risk identification to precise, individualized interventions and fully personalized medicine.

## 4 Clinical management strategies and challenges

The comorbid presence of MAFLD and CHB substantially increases HCC risk and poses challenges for clinical management across multiple domains, including screening, diagnosis, and treatment (56). The primary management difficulty lies in their complex interplay throughout the disease course. Early-stage hepatic steatosis may transiently suppress HBV replication, whereas prolonged metabolic disturbances like steatohepatitis accelerate liver fibrosis, synergistically amplifying HCC risk (26, 57, 58). This bidirectional pathophysiology necessitates a dual-track clinical strategy addressing both virological control and metabolic health (11, 21).

Early identification and precise risk stratification of CHB–MAFLD comorbidity are essential. Routine MAFLD screening is recommended for all CHB patients, including assessment of hepatic steatosis via imaging or biomarkers and systematic evaluation of metabolic risk factors. Importantly, even with effective viral suppression, metabolic comorbidities can continue to drive progression. Moreover, the number of metabolic abnormalities correlates with HCC risk in a dose-dependent manner (21, 59). Current HCC surveillance strategies are still largely based on traditional risk factors such as cirrhosis and chronic viral hepatitis. However, whether existing surveillance recommendations are sufficient for non-cirrhotic CHB–MAFLD patients remains uncertain. Emerging evidence suggests that metabolic dysfunction and steatohepatitis may contribute to hepatocarcinogenesis even in the absence of advanced fibrosis or cirrhosis (7, 17, 18). Accordingly, surveillance strategies may need to be further individualized or intensified in selected non-cirrhotic CHB–MAFLD populations with a high metabolic risk burden, although prospective evidence is still needed (21, 59). Beyond surveillance, management objectives must also extend beyond achieving virological response to actively addressing metabolic disorders. For CHB, guideline-recommended potent antiviral agents should be administered, with attention to potential comorbidity impacts on drug efficacy (e.g., Entecavir) necessitating enhanced monitoring. For MAFLD, lifestyle interventions remain the cornerstone, aiming to optimize metabolic parameters through weight control. While no specific pharmacotherapy is currently approved, systematic management of blood glucose, lipids, and blood pressure provides hepatoprotective benefits. Evidence indicates that diabetes and central obesity are independent HCC risk factors in CHB patients, highlighting the importance of targeted interventions (60, 61).

Current management of CHB–MAFLD comorbidity faces three principal challenges. First, risk stratification remains imprecise. Due to substantial MAFLD heterogeneity, the effects of hepatic steatosis and metabolic dysfunction on prognosis can be opposing, complicating quantification of their individual contributions and hindering personalized monitoring strategies (19). Second, a potential treatment paradox exists: it remains unclear whether reducing hepatic fat might diminish its transient suppressive effect on HBV, potentially precipitating viral rebound. Accordingly, intensified monitoring of virological markers and liver function is recommended during initial metabolic intervention (13). Third, robust evidence-based guidance is limited. Current recommendations largely derive from retrospective studies, with a paucity of prospective clinical trial data specifically for this comorbidity. Moreover, CHB patients are frequently excluded from trials of novel MAFLD pharmacotherapies, leaving their safety and efficacy in this population uncertain (11, 23).

In summary, managing CHB–MAFLD comorbidity has entered a new phase that equally prioritizes antiviral therapy and metabolic management. Future efforts should focus on prospective research to elucidate mechanistic interactions and generate high quality, targeted clinical evidence. Such advances will enable a shift from passive, reactive management to proactive, intervention-oriented strategies, effectively improving patient outcomes.

## 5 Discussion and perspectives

CHB–MAFLD comorbidity represents a central research frontier in hepatology, given its complex impact on HCC risk through diverse mechanisms and predictive challenges. Its biological and clinical manifestations are highly dynamic across molecular, clinical, and prognostic dimensions. Systematic study of this condition will elucidate HCC pathogenesis and offer a theoretical and practical framework for early risk identification and intervention.

A key implication of this review is that simple steatosis and NASH should not be treated as equivalent biological or clinical entities in CHB–MAFLD comorbidity. This distinction is important for future risk prediction and surveillance strategies, which should incorporate refined metabolic phenotyping rather than considering MAFLD as a uniform risk modifier.

Mechanistically, the interaction between CHB and MAFLD is dynamic and stage-dependent. Early metabolic activation may transiently suppress HBV replication, whereas progressive steatohepatitis and metabolic dysfunction promote fibrosis and hepatocarcinogenesis through lipotoxicity, oxidative stress, mitochondrial injury, and HBx-related oncogenic signaling. This explains the persistently high HCC risk despite viral suppression and underscores that antiviral therapy must be combined with metabolic management.

Traditional HCC risk models are linear and population-specific, failing to capture the non-linear interplay between metabolic and viral factors or include key metabolic indicators. Newer models offer better prediction but are limited by narrow variable selection and lack of broad validation. While ML/AI can model complex risk patterns for personalized prediction, their clinical use is hindered by poor generalizability, low interpretability, and non-standardized data. Currently, no HCC risk model is tailored to the CHB–MAFLD population, an urgent unmet clinical need.

Future clinical management of CHB–MAFLD comorbidity requires an integrated strategy combining antiviral and metabolic interventions. Centered on sustained, potent HBV suppression, this approach should incorporate systematic MAFLD screening followed by risk-stratified management based on metabolic dysfunction severity. Weight control and glycemic optimization are essential to mitigate steatohepatitis, restore hepatic metabolic homeostasis, and substantially reduce long-term HCC risk. Effective management requires collaboration across hepatology, endocrinology, oncology, and data science. The overarching aim is to establish an integrated framework for prevention, early detection, intervention, and surveillance, supported by biomarkers and risk-prediction models. This approach shifts the focus from etiological treatment to risk-based prevention, ultimately improving outcomes and reducing the burden of liver cancer.
